# A study of photomodulated reflectance on staircase-like, n-doped GaAs/Al_*x*_Ga_1−*x*_As quantum well structures

**DOI:** 10.1186/1556-276X-7-622

**Published:** 2012-11-12

**Authors:** Omer Donmez, Ferhat Nutku, Ayse Erol, Cetin M Arikan, Yuksel Ergun

**Affiliations:** 1 Department of Physics, Faculty of Science, Istanbul University, Vezneciler, Istanbul, 34134, Turkey; 2 Department of Physics, Faculty of Science, Anadolu University, Eskisehir, 26470, Turkey

**Keywords:** Photomodulated reflectance, Quantum well infrared photodetectors (QWIP), Aspnes’ third derivative form, Excitonic levels., 85.30.De, 85.60.-q, 71.55.Eq.

## Abstract

In this study, photomodulated reflectance (PR) technique was employed on two different quantum well infrared photodetector (QWIP) structures, which consist of n-doped GaAs quantum wells (QWs) between undoped Al_*x*_Ga_1−*x*_As barriers with three different *x* compositions. Therefore, the barrier profile is in the form of a staircase-like barrier. The main difference between the two structures is the doping profile and the doping concentration of the QWs. PR spectra were taken at room temperature using a He-Ne laser as a modulation source and a broadband tungsten halogen lamp as a probe light. The PR spectra were analyzed using Aspnes’ third derivative functional form.

Since the barriers are staircase-like, the structure has different ground state energies; therefore, several optical transitions take place in the spectrum which cannot be resolved in a conventional photoluminescence technique at room temperature. To analyze the experimental results, all energy levels in the conduction and in the valance band were calculated using transfer matrix technique, taking into account the effective mass and the parabolic band approximations. A comparison of the PR results with the calculated optical transition energies showed an excellent agreement. Several optical transition energies of the QWIP structures were resolved from PR measurements. It is concluded that PR spectroscopy is a very useful experimental tool to characterize complicated structures with a high accuracy at room temperature.

## Background

Quantum well infrared photodetector (QWIP) structures have been developed since 1990s [1]. There are many different types of QWIP structures. QWIPs can be categorized by their electrical properties: photovoltaic or photoconductive, or by their layer thicknesses: multi-quantum wells (MQW) or superlattice structures. They can also be categorized by having optical responsivity at a single or multiple wavelengths. Multi-color QWIPs can be composed of double barriers [2], stepped quantum wells [3], and stepped barriers. The structures with stepped barriers are also called as staircase-like QWIPs in the literature [4].

In this work, photomodulated reflectance (PR) and photoluminescence (PL) experiments were carried out on two different staircase-like QWIP structures at room temperature. PR is a powerful characterization method to determine optical transitions in both bulk and low-dimensional multilayer semiconductor structures. Its absorption-like character and high sensitivity makes it possible to observe optical transitions between ground and excited states, even at room temperature. PR spectroscopy utilizes the modulation of the built-in electric field at the semiconductor surface or at the interfaces through photo-injection of electron–hole pairs generated by a chopped incident laser beam. This technique produces sharp spectral features related to the critical points of the band structure. This provides a more explicit comparison of experimental results with theoretical models. However, PL only gives information about ground state transitions in QWs at room temperature. PR spectra were analyzed using the third derivative functional form (TDFF) in order to fit the optical transition energies, and the results were compared to the theoretical values calculated using transfer matrix method.

### Theory

Transfer matrix technique is a common method for solving Schrödinger equation for MQW structures which consist of layers having different band gaps and effective masses. By virtue of this technique, energy levels, wave functions under zero or constant electric field can be calculated in complex structures [5-7]. In this work, we had employed this technique to calculate the energy levels in each QW at 300 K.

In order to determine the band gap of GaAs at room temperature, Varshni equation [8] was used:

(1) $$E_{g}\left(T\right)=E_{g}\left(0\right)-\frac{\alpha T^{2}}{T+\beta }\text{,}$$

where *E*_g_(0) is the band gap of GaAs at *T* = *0* K; *α* = 5.405 × 10^−4^ eV/K and *β* = 204 K are Varshni parameters at the *Г* point. For Al_*x*_Ga_1−*x*_As ternary alloys. Temperature dependence of the band gap for *x* <*0*.*4* can be estimated by:

(2) $$E_{g}\left(x,T\right)=1.519+1.155x+0.37x^{2}-\frac{\alpha T^{2}}{T+\beta }\text{,}$$

where *α* and *β* are Varshni parameters of Al_*x*_Ga_1−*x*_As. Adachi showed that compositional dependence of Varshni parameters becomes significant in Al_*x*_Ga_1−*x*_As ternary alloys for *x* > 0.4 [9]. However, since *x* < 0.4 for Al_*x*_Ga_1−*x*_As in our structures, we used the same values as GaAs [9,10]. The conduction and the valance band offsets were chosen as 60% and 40%, respectively. In the calculations of energy levels, the effective mass for each layer was considered separately. The effective masses of electrons in AlAs and GaAs were taken as 0.15 and 0.067, respectively. Using these values, the effective mass of electrons in Al_*x*_Ga_1−*x*_As layers was calculated by applying Vegard’s law:

(3) $$m_{{\text{Al}}_{x}\text{Ga}{\;}_{1-x}\text{As}}=\frac{m_{\text{AlAs}}\;m_{\text{GaAs}}}{x\;m_{\text{GaAs}}+\left(1-x\right)m_{\text{AlAs}}}\text{.}$$

Similarly, the effective masses of holes in the Al_*x*_Ga_1−*x*_As layers were also calculated using Equation 3, taking the density of states heavy hole effective masses as 0.81 and 0.55, and the averaged light hole effective masses were taken as 0.16 and 0.083 in AlAs and GaAs, respectively [9].

PR spectra were fitted using the linear combination of several Aspnes’ TDFFs [11], expressed as:

(4) ΔRR=Re∑j=1nAje−iφjE−Egj+iΓj−mj+fjE,

where *n* is the number of spectral features to be fitted; *E* is the photon energy; *A*_*j*_, *φ*_*j*_, *E*_*gj*_, and *Г*_*j*_ are the amplitude, phase, band gap energy, and line broadening of the *j*_*th*_ feature, respectively. *m*_*j*_ represents the type of critical point depending on the dimensionality of the structure, and its value is 2.5 or 3 for 3-D (bulk) or 2-D cases, respectively. The background signal in the measurements was simulated and suppressed from Equation 4 by a linear *f*(*E*) function.

## Methods

PR and PL measurements were carried out on two different MQW structures at room temperature. A tunable monochromatic probe light was provided by a 100-W tungsten lamp, dispersed by a single grating monochromator, and the sample was pumped with a modulated 10-mW He-Ne laser at 632.8 nm that was mechanically chopped at 280 Hz. The reflected probe beam was measured by a Si photodiode. The AC and DC components of reflectance (R) and differential changes in *R* (*ΔR*) were acquired by a computer, simultaneously.

**Figure 1:**
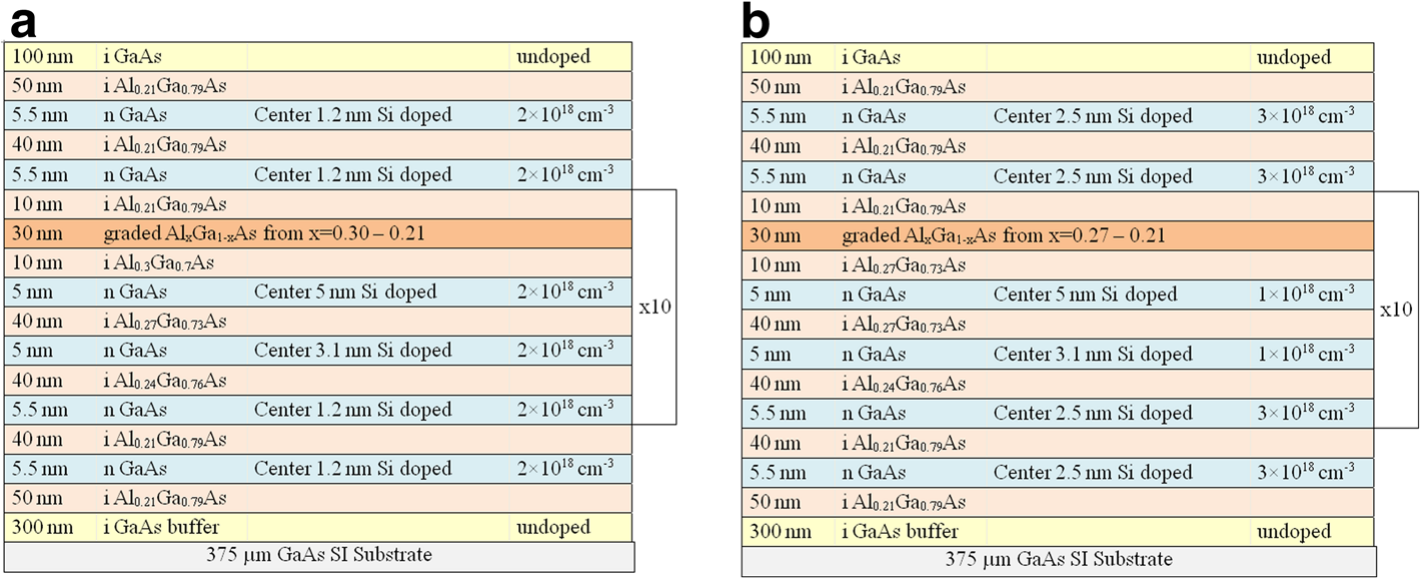
Schematic layer structure of (a) ANA14 and (b) IQE14 sample.

**Figure 2:**
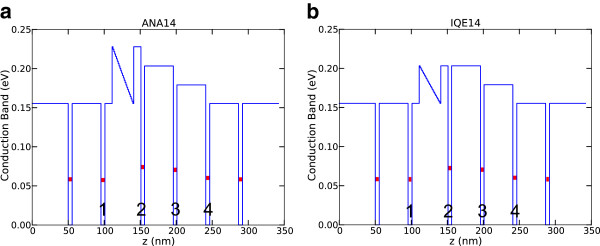
**Conduction band profile.** (**a**) ANA14 and (**b**) IQE14 structures. One period of the active region is shown in the diagram.

The structures used in this study consist of n-doped GaAs QWs sandwiched between undoped Al_*x*_Ga_1−*x*_As barriers with three different *x* compositions, producing staircase-like barriers. The structures were designed as QWIP devices. Details of the structures are given in Figure 1. The main differences between the two structures are the doping profile, the doping concentration, and the barrier composition of the triangular well. All QWs in ANA-coded samples have a doping concentration of 2 × 10^18^ cm^−3^. On the other hand, in IQE samples, QWs with 5.5- and 5-nm well width have doping concentrations of 3 × 10^18^ and 1 × 10^18^ cm^−3^, respectively. QWs with central doping width of 1.2 nm in the ANA structure were replaced by 2.5 nm in IQE structure. ANA samples have triangular wells in the active period with barriers containing 30% Al concentration. Besides, IQE-coded samples have 27% Al concentration. One edge of the triangular quantum well is formed by a graded barrier; the other edge is formed by a fixed barrier as seen from the potential profile of the conduction band given in Figure 2. We have introduced the graded barrier in the structure in order to provide a quasi-electric field which facilitates the drift current of the photo-excited carriers to adjacent layers. In Figure 2, quantum wells which have different barrier heights are labeled with numbers.

## Results and discussion

**Figure 3:**
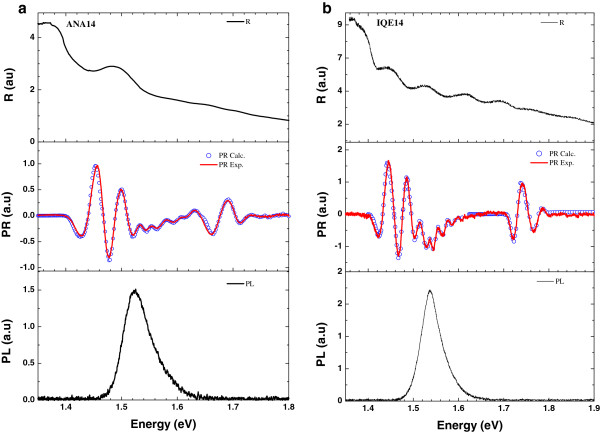
**R, PL, and PR spectra.** (**a**) ANA14 and (**b**) IQE14 sample. Red line is the experimental, while open circles are the calculated PR spectra points.

Experimental results on reflectivity (R), PL, and PR spectra of ANA14 and IQE14 structures are given in Figure 3. Calculated PR spectra are also included in the figure. The R spectra are given just for information and not to be included in the discussion. PL signal begins to rise from the fundamental band edge of bulk GaAs and peaks at about the combined excitonic transition region. Details of the excitonic transitions are smeared out. However, in the PR spectra, the fundamental band gaps of bulk GaAs cap layer and Al_*x*_Ga_1−*x*_As barrier regions, and a series of excitonic transitions are clearly resolvable. In order to analyze the obtained PR spectra, we divided the spectrum into three regions. The first region between 1.4 to 1.51 eV includes signals from the bulk GaAs and effective band gap due to e1-hh1 excitonic transitions of doped GaAs QWs having Al_*x*_Ga_1−*x*_As barriers with *x* = 0.21. The second region ranging from 1.51 to 1.6 eV includes the other excitonic transitions such as e1-hh1, e1-hh2, and e1-lh1, coming mainly from the active period of the structures. Finally, 1.6 to 1.8 eV region includes PR signals of Al_*x*_Ga_1−*x*_As layers. The experimental results exhibit Lorentzian-like peaks which are obtained from Equation 4 as the modulus of PR resonances according to the equation below:

(5) $$\left|\Delta \rho \right|=\frac{A}{{\left[{\left(E-E_{0}\right)}^{2}+\Gamma^{2}\right]}^{m/2}}\text{.}$$

Using this equation, bulk and excitonic transition parameters *A* and *Γ* of each signal were determined. These parameters were placed into Equation 4, and then the optical transition energies were calculated. The calculated energy levels and corresponding PR peaks in the spectra are summarized in Table 1. All possible excitonic transitions in the calculated PR spectra are clearly distinguishable. Experimental and calculated PR spectra are in excellent agreement. Although most of the excitonic transitions are identified, some of the calculated transitions were not observed in the PR spectra (Table 1).

**Table 1:** **Electron and hole energy levels and PR peaks of the QWIP structure obtained at *T *= 300 K**

**Structure name**	**QW number**	**Well width (nm)**	**e**_**1**_**(meV)**	**hh**_**1**_**(meV)**	**hh**_**2**_**(meV)**	**lh**_**1**_**(meV)**	**E**_**g**_**(meV)**	**Effective *E***_**g**_**e**_**1**_**-hh**_**1**_**(meV)**	**PR peak e**_**1**_**-hh**_**1**_**(meV)**	**Effective *E***_**g**_**e**_**1**_**-hh**_**2**_**(meV)**	**PR peak e**_**1**_**-hh**_**2**_**(meV)**	**Effective *E***_**g**_**e**_**1**_**-lh**_**1**_**(meV)**	**PR peak e**_**1**_**-lh**_**1**_**(meV)**
ANA14	1	5.5	58.4	13.1	50.6	43.2	1.422	1.494	1.495	1.531	1.534	1.524	-
IQE14									1.490		-		
ANA14	2	5	74.1	16.3	63.4	55.1	1.422	1.513	-	1.560	-	1.552	1.550
													-
IQE14	2	5	72.5	16.1	62.5	53.8	1.422	1.511	-	1.557	-	1.549	-
													1.545
ANA14	3	5	70.5	15.9	61.4	52.2	1.422	1.509	-	1.554	1.550	1.545	-
IQE14									1.505		-		1.545
ANA14	4	5.5	60.2	13.4	51.6	44.6	1.422	1.496	-	1.534	-	1.527	-
IQE14											1.534		

PL studies showed just a single broad peak for ANA14 structure at 1.525 eV and for IQE14 structure at 1.539 eV at room temperature. As seen from the calculated values of the excitonic transitions in different quantum wells, the energy differences between them are quite small; therefore, the observed PL peak cannot be attributed to just one transition. It can be concluded that observed PL peak represents additive information about some of the optical transitions. However, PR provides detailed information, resolving closely separated energy levels, even at room temperature.

## Conclusion

The importance of the photomodulated reflectance spectroscopy in complicated semiconductor QW structures and hence in QWIPs has been verified by the experimental and the theoretical results obtained from this work. QWs with barriers having minor differences in the alloy composition can clearly be distinguished by PR measurements at room temperature. Indeed, e1-hh1, e1-hh2, and e1-lh1 transitions were clearly observed and resolved. On the other hand, in PL measurements, only one single photoluminescence peak was observed.

## Abbreviations

MQW: multi-quantum well; PL: photoluminescence; PR: photomodulated reflectance; QW: quantum well; QWIP: quantum well infrared photodetector; R: reflectance; TDFF: third derivative functional form.

## Competing interests

The authors declare that they have no competing interests.

## Authors’ contributions

OD carried out the experiments and fitted the PR spectra in collaboration with AE, MCA, and FN. FN calculated the energy levels. OD, FN, MCA, and AE contributed to the manuscript preparation. YE is the designer of the QWIP structure. All authors read and approved the final manuscript.
